# Real-Time In Vivo Imaging of Early Mucosal Changes during Ischemia-Reperfusion in Human Jejunum

**DOI:** 10.1371/journal.pone.0039638

**Published:** 2012-06-22

**Authors:** Joep Grootjans, Wim Hameeteman, Ad A. Masclee, Ronald M. van Dam, Wim A. Buurman, Cornelis H. C. Dejong

**Affiliations:** 1 Department of Surgery, NUTRIM School for Nutrition, Toxicology & Metabolism, Maastricht University Medical Center, Maastricht, The Netherlands; 2 Department of Gastroenterology, NUTRIM School for Nutrition, Toxicology & Metabolism, Maastricht University Medical Center, Maastricht, The Netherlands; Universidade de Sao Paulo, Brazil

## Abstract

**Background and study aims:**

Small intestinal ischemia-reperfusion (IR) is a frequent, potentially life threatening phenomenon. There is a lack of non-invasive diagnostic modalities. For many intestinal diseases, visualizing the intestinal mucosa using endoscopy is gold standard. However, limited knowledge exists on small intestinal IR-induced, early mucosal changes. The aims of this study were to investigate endoscopic changes in human jejunum exposed to IR, and to study concordance between endoscopic appearance and histology.

**Patients and methods:**

In 23 patients a part of jejunum, to be removed for surgical reasons, was isolated and selectively exposed to ischemia with 0, 30 or 120 minutes of reperfusion. In 3 patients, a videocapsule was inserted in the isolated segment before exposure to IR, to visualize the mucosa. Endoscopic view at several time points was related to histology (Heamatoxylin & Eosin) obtained from 20 patients.

**Results:**

Ischemia was characterized by loss of villous structure, mucosal whitening and appearance of punctate lesions. This was related to appearance of subepithelial spaces and breaches in the epithelial lining in the histological view. Early during reperfusion, the lumen filled with IR-damaged, shed cells and VCE showed mucosal erosions, hemorrhage and intraluminal debris. At 60 minutes of reperfusion, the only remaining signs of IR were loss of villous structure and small erosions, indicating rapid mucosal healing.

**Conclusions:**

This study shows a unique, real-time in vivo endoscopic view of early mucosal changes during IR of the human small intestine. Future studies should evaluate its usefulness in diagnosis of patients suspected of IR.

## Introduction

Small intestinal ischemia is a frequently occurring, life threatening phenomenon. It arises from occlusion, vasospasms and/or hypoperfusion of the mesenteric vasculature. Mortality rates exceed 60% and have remained stable over the past decades. [1], [2] Mortality rates are linked to the development of transmural damage, and translocation of bacteria to the circulation, causing systemic inflammation and multiple organ failure. [3], [4] In addition, a variety of studies showed a link between intestinal IR and the development of acute lung injury (ALI), which strongly contributes to the high mortality. [5], [6] Early recognition of mesenteric ischemia has been shown to significantly improve patient outcome, [7] however, timely decision making is challenging, since clinical presentation is often nonspecific and there is a lack of diagnostic tests to assess intestinal damage. In addition, diagnosis often requires invasive testing, including angiography, exposing patients who typically have several comorbidities to risk. [1], [2].

Although colonoscopy has already been implemented in the diagnostic workup of patients suspected of colonic ischemia, upper endoscopy has little place in the diagnosis of small intestinal ischemia. [8] One downside of upper endoscopy is that detection of mesenteric ischemia is limited by the reach of the instrument, which makes it difficult to trace ischemic areas in distal parts of the small intestine. In contrast, chronic mesenteric ischemia often also involves the proximal part of the small intestine, and recent advances in endoscopic techniques such as visible light spectroscopy, have been shown to be valuable in diagnosing chronic mesenteric ischemia. [9], [10] A second potential drawback of gastrointestinal endoscopy in the detection of mesenteric ischemia, is that endoscopists should avoid intraluminal pressures exceeding 30 mmHg, since this has been shown to decrease blood flow in the small bowel and colon. [8], [11] Moreover, care should be taken to avoid overinflation or advancement of the scope beyond the affected area to minimize the risk of bowel perforation.

An approach that could circumvent these problems is the use of videocapsule endoscopy (VCE). Since the introduction of VCE by Iddan et al. in *Nature* in 2000, [12] the device is increasingly used to non-invasively visualize the gastrointestinal mucosa in a variety of diseases. In this study, we set out to investigate real-time in vivo mucosal changes during the early phases of human small intestinal ischemia-reperfusion, by using VCE technology in a newly developed human experimental IR-model. [13], [14] In addition, we provide the relation between endoscopic view and histological appearance of human jejunum exposed to IR.

## Materials and Methods

### Ethics

The study was approved by the Medical Ethical Committee of Maastricht University Medical Center and was conducted according to the revised version of the Declaration of Helsinki (October 2008, Seoul). All patients were informed about the experimental procedures and written informed consent of all patients was obtained prior to the surgical procedure.

### Patients and Surgical Procedures

#### Patients

23 patients undergoing pancreatico-duodenectomy were included in this study. VCE data were obtained from 3 patients included in our newly developed human intestinal IR model, and VCE data were compared to histology from 20 patients included in the same human intestinal IR model.

#### Human intestinal IR protocol

**Figure 1:**
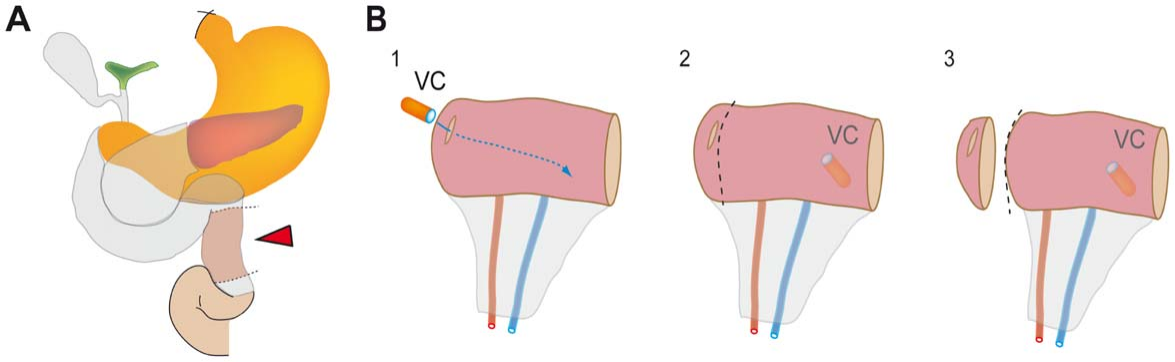
Human experimental model to study real time in vivo mucosal changes during human intestinal IR. (A) During a pylorus-preserving pancreaticoduodenectomy, a part of otherwise healthy intestine is removed for surgical reasons. A small segment (red arrowhead) of jejunum is isolated on both sides using a cutting stapler, allowing us to selectively expose it to ischemia and reperfusion. (B) Prior to induction of ischemia, a small bowel videocapsule is introduced into the isolated part of intestine via a small incision and positioned at the distal end of the isolated part of intestine (1 and 2). The part with the incision is resected using a cutting stapler (3), to rule out any possible leakage from intraluminal content towards the abdominal cavity.

The experimental protocol was performed as previously described. [13], [14] During pancreatico-duodenectomy, a variable length of jejunum is routinely resected in continuity with the head of the pancreas and duodenum as part of the surgical procedure (Figure 1A). The terminal 6 cm of this jejunal segment was isolated and subjected to either 30 or 60 minutes of ischemia by placing two atraumatic vascular clamps across the mesentery (30I: n = 10, 60I: n = 13, of which 3 patients were included in the VCE protocol (see below)). Meanwhile, surgery proceeded as planned. After ischemia, one third (2 cm) of the isolated ischemic jejunum was resected using a linear cutting stapler (GIA™, Covidien, Mansfield, MA). Next, clamps were removed to allow reperfusion, as confirmed by regaining of normal pink color and restoration of gut motility. Another segment of the isolated jejunum (2 cm) was resected similarly after 30 minutes of reperfusion, and 120 minutes of reperfusion. Simultaneously, 2 cm of jejunum which remained untreated during surgery was resected and served as internal control tissue. Tissue samples were immediately formalin-fixed.

#### VCE protocol

To visualize the mucosa during IR, a small bowel VC (Pillcam™-SB2, Given Imaging, Yokneam, Israel) was inserted in the isolated part of intestine of 3 patients (2F:1M, mean age 64y), prior to the induction of ischemia (Figure 1B). The videocapsule was inserted via a small incision (see Figure 1B), and after insertion of the capsule, the piece of jejunum with the incision was stapled off, to prevent any possible leakage of intraluminal content towards the abdomen (see Figure 1B). After recording the endoscopic view of healthy mucosa, the human intestinal IR procedure was initiated (see detailed protocol above). VCE data were investigated using RapidReader v6.3 software (Given Imaging), and endoscopic view was captured at several time points (30I, 60I, 60I 30R, 60I >60R). Macroscopic changes were compared to the histological appearance in Haematoxylin&Eosin (H&E) staining from jejunum exposed to 30I (n = 10 patients, 7M:3F), or 60I with 0R, 30R and >60R (n = 10 patients, 8M:2F). [14], [15].

### Histology

Tissue specimens obtained during the experimental protocol were immediately immersed in 4% formaldehyde fixative (Unifix, Klinipath, Duiven, the Netherlands) and incubated overnight at room temperature. Next, tissue samples were embedded in paraffin and 4 µm sections were cut. For morphological analysis, sections were deparaffinized in xylene and rehydrated in graded ethanol to distilled water and stained with Haematoxylin&Eosin (H&E).

## Results

### Human Intestinal Ischemia Induces Pale Discoloration of the Mucosa and Small Punctate Lesions

**Figure 2:**
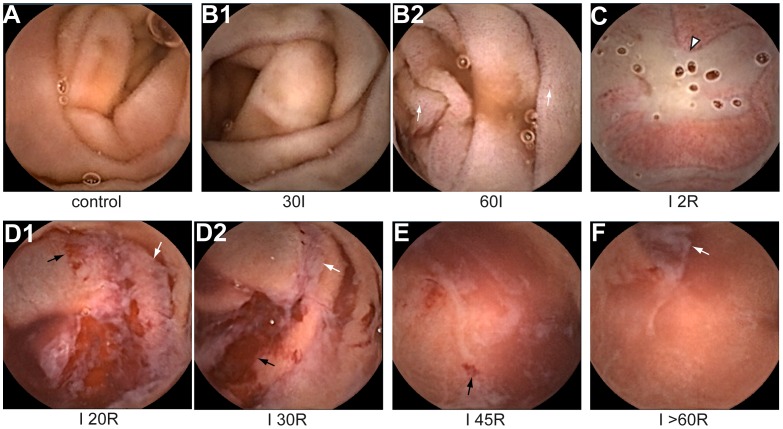
Endoscopic appearance of human jejunum exposed to IR. (A) Normal endoscopic appearance of control tissue (B1) Pale discoloration of jejunal mucosa after 30 minutes of ischemia (B2) Appearance of small punctate lesions (white arrows) within the pale mucosa at 60 minutes of jejunal ischemia. (C) Within a few minutes of reperfusion, the intestinal lumen fills with a white substance (white arrowhead). (D1–D2) At 20–30 minutes of reperfusion, villous architecture in the mucosa is loss, and areas with hemorrhage (black arrows) and superficial erosions become apparent. Note the ‘white clouds’ of debris in the intestinal lumen (white arrows). (E) At 45 minutes of reperfusion, areas with hemorrhage and erosion are reduced. (F) At 60 minutes of reperfusion, the mucosa regained its initial color. Remaining signs of ischemia are the spots with hemorrhage, the intraluminal debris (white arrow), and loss of the villous structure.

**Figure 3:**
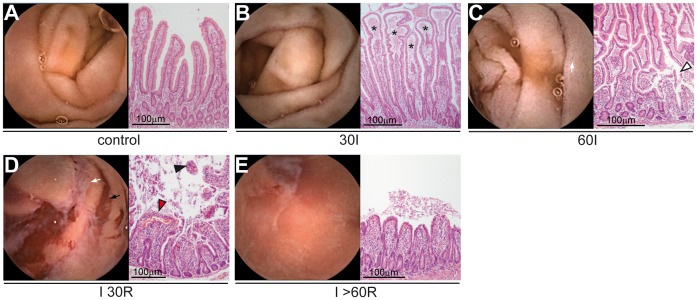
Concordance between endoscopic and histological appearance. (A) Normal endoscopic view and histological appearance. (B) At 30 minutes of ischemia, subepithelial spaces appear (asterisks), indicating retraction of the basement membrane. This relates to the pale discoloration of mucosa in the endoscopic view. (C) At 60 minutes of ischemia, breaches in the epithelial lining can be observed (arrowhead), which relate to the appearance of punctate lesions in the endoscopic view (white arrow). (D) At 30 minutes of reperfusion, hemorrhage and shedding of IR-damaged epithelial cells becomes evident in both histological view (right panel: red arrowhead and black arrowhead, respectively), and endoscopic view (left panel: black arrow and white arrow, respectively). (E) At 60 minutes of reperfusion, villus atrophy in observed in conjunction with gaps in the epithelial lining and luminal debris. This relates to the loss of villous structure in endoscopic view, and the ‘white clouds’ of intraluminal debris.

Before induction of ischemia, jejunal mucosa appeared healthy as indicated by the pink color and villous structure (Figure 2A). H&E staining showed normal histological appearance (Figure 3A), indicating good accordance between macroscopic view and histology. During ischemia, the mucosa gradually became pale (Figure 2B). The villous structure had disappeared at 30 minutes of ischemia, although signs of macroscopic mucosal damage were absent (Figure 2B1). At this point, histology showed retraction of the basement membrane leading to subepithelial spaces (Figure 3B), which might explain the loss of villous structure in the endoscopic view. Even after 60 minutes of ischemia, macroscopic mucosal damage was limited to punctate lesions and focal spots of hemorrhage within the white-appearing mucosa (Figure 2B2). In histology, these punctate lesions appeared to arise from detachment of epithelial sheets (Figure 3C).

### Early Reperfusion of Ischemic Intestine is Associated with Development of Intraluminal Debris, Mucosal Erosions and Hemorrhage

To study the consequences of reperfusion of ischemic jejunum, vascular clamps were removed after 60 minutes of ischemia. Interestingly, the intestinal lumen rapidly filled with a milky substance (Figure 2C), while the underlying mucosa exhibited signs of erosion (Figure 2C). After 15–20 minutes of reperfusion, the intraluminal debris was aspirated by needle puncture of the intestinal segment to regain vision on the mucosa. At this point, hemorrhage became apparent and large regions showed loss of the typical small intestinal villous structure (Figure 2D1). At 30 minutes of reperfusion, parts of the mucosa had regained color, while sharply demarcated regions showed superficial ulceration and hemorrhage (Figure 2D2). White ‘clouds’ of intraluminal debris were observed, which represented shed IR-damaged cells together with fluids and blood in H&E staining (Figure 3D). [16].

### IR-damaged Mucosa is Rapidly Restored during Reperfusion

Remarkably, this heavily damaged mucosa recovered rapidly, since within 45 minutes of reperfusion, the areas with hemorrhage and erosions were reduced (Figure 2E), and after 60 minutes of reperfusion the mucosa had regained its initial color (Figure 2F). The remaining signs of ischemia were small erosions, spots with hemorrhage, intraluminal debris, and loss of villous structure (Figure 2F). Histologically, villi were strongly reduced in length. In addition, the epithelial lining was recovering in most of the villi, while IR-damaged shed epithelial cells were observed in the intestinal lumen (Figure 3E).

## Discussion

In this study, VCE was used in a recently developed human experimental IR model, to visualize for the first time the early mucosal changes during IR in man. First, this study demonstrates that mucosal changes in the early phase of intestinal ischemia can be limited to a pale discoloration of the mucosa, with appearance of small punctate lesions when ischemia progresses. Second, we show that mucosal IR-induced damage occurs particularly during the reperfusion phase. Third, we show that the human small intestine has a remarkable capacity to recover from ischemia, since remaining signs of ischemia at 60 minutes of reperfusion were limited to loss of villous structure, small erosions, and presence of intraluminal debris.

In recent years, VCE is increasingly used for non-invasive detection of mucosal pathology throughout the whole intestinal tract. [17] In two case reports, VCE was successfully used to detect acute mesenteric ischemia with bowel necrosis, [18] and diffuse ischemia in a patient with Wegener’s granulomatosis. [19] In both these cases, ischemia was characterized by ulceration, erythema, edema, bleeding, and patchy necrotic areas. Here, we show that VCE can also detect very early IR-induced changes to the small intestinal mucosa, which are mainly characterized by whitening of the mucosa and appearance of small punctate lesions. This suggests that VCE could help in decision making during the diagnostic workup. Moreover, VCE could be useful to localize ischemia in parts of the intestine that are difficult to visualize with routine endoscopy, which could be used in guiding the surgeon to surgical intervention. However, the limited changes during early ischemia emphasize the importance of clinical suspicion and alertness during endoscopy/VCE in patients.

Next to the mucosal changes following ischemia, we demonstrate that reperfusion is associated with massive mucosal damage to the epithelial lining. Previous studies have also shown that on a histological level, reperfusion of ischemic intestine causes massive cell shedding at the villus tips. [16], [20], [21] In addition, the reperfusion phase was associated with apoptosis of specialized Paneth cells in the crypts, [14] which play a role in immunological intestinal barrier function. Both these events facilitate bacterial translocation and inflammation, [14], [21] and it is therefore important to bear in mind that, although rapid restoration of the blood flow is imperative to improve patient outcome, reperfusion paradoxically induces extensive intestinal barrier loss.

In addition to the death cells that are observed in the intraluminal ‘milky’ substance, it is also likely that this substance contains mucus, either secreted from goblet cells, or as a consequence of goblet cell loss. We have recently shown that ischemia with and without reperfusion in the human and rat colon is associated with detachment of the mucus layer from the epithelium and increased secretion of goblet cell mucus into the intestinal lumen. [22] The rapid increase in intraluminal mucus contents, accompanied by the hemorrhage as observed in this study, explains why patients suffering from intestinal ischemia can have mucous and bloody stools. In addition, this pathophysiological event might lead to further complications, since loss of the mucus layer in the small intestine has been linked to decreased protection to intraluminal bacteria and pancreatic enzymes. [23] This increases tissue damage and bacterial translocation to the internal milieu. [24] Together, both death cells and intruding pathogens are likely to cause an inflammatory response following intestinal IR, characterized by neutrophil influx and expression of inflammatory cytokines. [14], [21], [25], [26] This will eventually also trigger inflammation in distant organs including the lung, which highly contributes to intestinal IR-induced morbidity and mortality. [4], [5], [6], [27].

Unfortunately, there are at present only limited possibilities to prevent reperfusion-induced inflammatory complications with the current treatment modalities. It is therefore tempting to speculate on possible future preventive strategies using the VCE. For example, recent evidence shows that endoplasmic reticulum (ER) stress is an important pathophysiological phenomenon in human intestinal IR, associated with aforementioned Paneth cell apoptosis and increased bacterial translocation and inflammation. [14] Topical intraluminal treatment with agents that alleviate ER stress, including the FDA approved tauroursodeoxycholic acid (TUDCA) and 4-phenyl butyrate acid (PBA), could be a potential future preventive of therapeutic strategy to reduce IR-induced complications. In addition, intraluminal release of anti-inflammatory agents could dampen the local inflammatory response as well as the subsequent tissue damage, thereby preventing bacterial translocation, excessive inflammation, and potentially also distant organ injury. Topical treatment using VCE is feasible in the foreseeable future, since new technology has been developed that allows maneuvering of the capsules in the intestine (via magnets or propellers). [28], [29] This can halt the capsule at a desired spot to release topical agents. In addition, such techniques could prevent capsule retention when bowel movements are reduced as a consequence of pathological situations, including intestinal ischemia.

In conclusion, we have provided a first unique, real-time in vivo view of the early mucosal changes during IR of the human small intestine. Future studies should be directed at evaluating the usefulness of VCE as part of the diagnostic workup of patients suspected of splanchnic ischemia. Moreover, VCE might be used to topically treat IR-induced damage, or limit reperfusion-induced damage after restoration of the blood flow.
